# Automatic extraction of endocranial surfaces from CT images of crania

**DOI:** 10.1371/journal.pone.0168516

**Published:** 2017-04-13

**Authors:** Takashi Michikawa, Hiromasa Suzuki, Masaki Moriguchi, Naomichi Ogihara, Osamu Kondo, Yasushi Kobayashi

**Affiliations:** 1 Center for Environmental Innovation Design for Sustainability, Osaka University, Suita, Osaka, Japan; 2 Department of Precision Engineering, The University of Tokyo, Bunkyo, Tokyo, Japan; 3 Department of Information and System Engineering, Chuo University, Bunkyo, Tokyo, Japan; 4 Department of Mechanical Engineering, Keio University, Yokohama, Kanagawa, Japan; 5 Department of Biological Sciences, The University of Tokyo, Bunkyo, Tokyo, Japan; 6 Department of Anatomy, National Defense Medical College, Tokorozawa, Saitama, Japan; Chinese Academy of Sciences, CHINA

## Abstract

The authors present a method for extracting polygon data of endocranial surfaces from CT images of human crania. Based on the fact that the endocast is the largest empty space in the crania, we automate a procedure for endocast extraction by integrating several image processing techniques. Given CT images of human crania, the proposed method extracts endocranial surfaces by the following three steps. The first step is binarization in order to fill void structures, such as diploic space and cracks in the skull. We use a void detection method based on mathematical morphology. The second step is watershed-based segmentation of the endocranial part from the binary image of the CT image. Here, we introduce an automatic initial seed assignment method for the endocranial region using the distance field of the binary image. The final step is partial polygonization of the CT images using the segmentation results as mask images. The resulting polygons represent only the endocranial part, and the closed manifold surfaces are computed even though the endocast is not isolated in the cranium. Since only the isovalue threshold and the size of void structures are required, the procedure is not dependent on the experience of the user. The present paper also demonstrates that the proposed method can extract polygon data of endocasts from CT images of various crania.

## Introduction

Understanding the causes and process of brain evolution in human lineage is a central problem in the field of physical anthropology. However, since soft tissues such as brain are not fossilized, endocasts must be analyzed in order to infer brain morphology enclosed in fossil crania. Although manually-replicated endocasts have long been used for the materials [1], recent developments in virtual anthropology have allowed to handle 3D CT images directly [2]. The primary objective of the present research is to extract endocasts as polygons.

The use of CT scanning technology is a promising approach for acquiring geometric data of crania. X-ray CT scanners can obtain cross-sectional images of the target objects, and 3D images of the target objects can be obtained by stacking the 3D images. The surface structure of the cranium obtained from CT images can be computed by isosurface extraction methods such as the Marching cubes algorithm [3].

Once the endocranial polygons are extracted, they can be used for various anthropological applications. The primary advantage of CT scanning of fossil crania is that the method provides non-destructive measurement. Thus, researchers can analyze crania in virtual space without the need for physical models. Virtual assembly of crania have been reported by several researchers [4–9]. Moreover, Amano et al. [10] reported decomposition and reassembly of the Neanderthal Amud 1 cranium in virtual space. Endocranial surfaces may also be used to identify cortical features from sulcus patterns imprinted on the surfaces [1, 11–13]. Endocranial models can also be used for variation analysis of brain morphology (e.g., [9, 14, 15]). Moreover, attempts have recently been made to reconstruct the brain morphology of Neanderthals by warping brains of modern humans based on endocranial morphology [16].

One of the primary issues in endocranial polygon extraction from CT images of crania is the requirement of manual operation. Since the endocranial surfaces exist inside the crania, other surfaces, such as exocranial surfaces, must be manually removed from their isosurfaces. This is a tremendous task because these surfaces are close to each other and manually removing exocranial surfaces sometimes results in the inadvertent removal of endocranial surfaces. An alternative approach is slice-by-slice contouring, which is relatively easy. However, tremendous tasks still exist, since the number of slices is usually large. Thus, endocast extraction becomes a bottleneck in digital anthropological research.

Extracting a meaningful region from the geometric data is known as segmentation. This is a classic problem in image processing and geometric modeling, and a number of segmentation methods have been investigated. Commonly used segmentation methods find a discontinuity in the intensity values or geometric features (e.g., curvature) using energy minimization problems such as active contours [17], the level set method [18], graph cut algorithms [19], and variants thereof. These methods work well for scanned images when proper parameter settings or energy functions are designed. However, the parameter settings used in these methods are complicated. For example, Liu et al. introduced a method for extracting human bones based on level set functions [20]. However, this method is designed only for extracting pole-like bones and is not efficient for extracting endocranial polygons. Michikawa et al. introduced a method for extracting vocal tracts based on mathematical morphology [21]. However, this method assumes that the extracted region is almost closed and extracting endocranial spaces that are largely open is difficult.

The present paper describes an automatic method for computing endocast shapes as polygonal data from CT images of human skulls. The proposed method is based on the observation that the endocranial region is the largest space in the skull. Based on this observation, the algorithm used in the proposed method is designed so that the background voxels are classified into the primary endocranial space and other smaller spaces. The proposed method consists of three major steps: binarization, segmentation, and polygonization. We first classify the CT images into the skull (foreground) and the background. Next, we extract the endocranial region from the input data based on watershed segmentation [22]. In this step, we first compute Euclidean distance fields from the binary images of the input data. The initial seed voxels of the endocranial region and other regions are assigned to the voxels with larger distance values. Segmentation is then performed by expanding the initial seed voxels based on the distance fields. Polygonization of the endocranial region is achieved by commonly used isosurface extraction methods [3] for the extracted region only.

One of the primary advantages of the proposed method is the automation of endocast extraction from human crania. The user needs only two parameters: the size of void structures (e.g. diploic space and cracks) and the isovalue threshold for binarization. This means that the extracted results are not dependent on the user’s experience. In addition, the proposed method can extract closed and manifold surfaces of endocranial polygons. This is efficient for various applications, including volume estimation and 3D printing, whereas manual operation requires time-consuming tasks such as hole filling and topological cleaning for generating completely closed manifold surfaces.

In the present study, we implemented the proposed method and applied the method to CT images of various types of crania, including fossil crania. The results demonstrate that the proposed method can automatically extract endocranial polygons from CT images.

## Method

**Fig 1:**
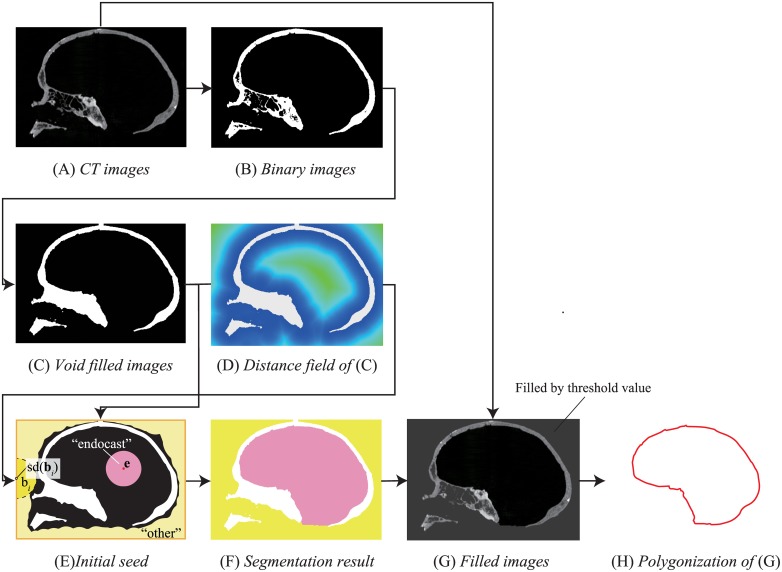
Overview of the proposed method. Note that the 2D images shown herein are cross-sections of 3D images.

Given CT images of crania, the proposed method computes endocranial polygons of crania. Fig 1 shows an overview of the proposed method. The proposed method consists of three major steps: binarization (Fig 1A–1C), segmentation(Fig 1C–1F), and polygonization (Fig 1F–1H).

The binarization step classifies the input CT image into voxels representing the cranium (foreground) and background voxels. We use a simple binarization with an appropriate threshold *t*, although other methods (e.g., automatic estimation by Otsu [23]) can also be used. Next, we apply the cavity detection method proposed in [24] based on a black (bottom) hat operator in mathematical morphology [25, 26] in order to remove small cavities in the cranium.

The segmentation step classifies background voxels in the binary image into endocast voxels and voxels of other types. The proposed method uses the watershed-based method [22] using distance field [27] of the binary images computed in the previous step. Given the binary image and its distance field, we first assign the initial seeds for the endocast voxels and other voxels (Fig 1E). Since the endocranial region is the largest empty space in the cranium, the center voxels of the endocast must have larger distance values. However, since the voxels with the largest distance values usually exist outside of the cranium, we first assign the “other” label to the edge voxels **b**_*i*_ (orange lines in Fig 1E) so that the center point of the endocast will be the voxel with maximum distance value. In addition, for each boundary voxel **b**_*i*_, we also assign the “other” label to its neighboring voxel **v**_*j*_ that satisfies ||**b**_*i*_ − **v**_*j*_|| ≤ *sd*(**b**_*i*_), where *d*(**b**_*i*_) denotes the distance at **b**_*i*_ and *s* denotes a scaling factor (*s* < 1) for assigning the label to the voxels with larger distance values. Since the voxel with the greatest distance from the rest of the voxels must be the center of the endocast voxels, we assign the “endocast” label to this voxel **e** and nearby voxels **v**_*j*_ that satisfy ||**e** − **v**_*j*_|| ≤ *sd*(**e**). Watershed segmentation is then applied to the binary images by expanding the initial seed voxels based on the distance field. When the expansion is stopped, background voxels are decomposed into the endocast voxels and other voxels.

The final step is polygonization of endocranial surfaces from CT images using an extended version of the partial polygonization method using mask images introduced in [21]. Prior to polygonization, We fill voxels with “other” label by the threshold value *t* used in the binarization step. This manipulation results in closed isosurfaces being obtained by the original Marching cubes algorithm.

## Results and discussion

### Results

We implemented the above algorithm as a Windows binary in C++. We used Eigen library [28] for linear algebra computation in our implementation, and other parts are developed from scratch. We also applied the algorithm to various types of crania, as summarized in Table 1.

**Table 1:** Data sets.

Specimen ID	Description	Size	Voxel size[mm]	Data courtesy
Mladec1	Fossil human	512x512x360	0.47x0.47x0.47	Univ. of Vienna
KUMA3008	Modern Japanese	512x512x299	0.47x0.47x0.5	Laboratory of Physical Anthropology, Kyoto Univ. [15]
KUMA3147	Modern Japanese	512x512x301	0.47x0.47x0.5	Laboratory of Physical Anthropology, Kyoto Univ. [15]
M15	Crab-eating monkey	512x512x253	0.35x0.35x0.5	Kyorin Univ./National Defense Medical College [13]
M16	Crab-eating monkey	512x512x257	0.35x0.35x0.5	Kyorin Univ./National Defense Medical College [13]
CT Head	Human cadaver	512x512x113	1 x 1 x 2	Univ. of North Carolina

**Fig 2:**
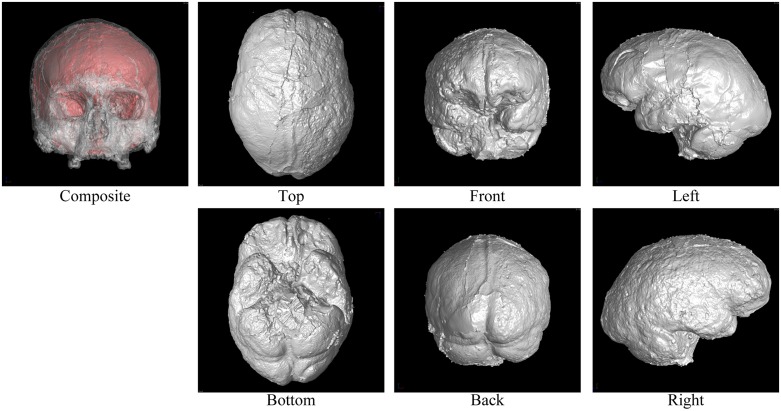
Results of endocast extraction (Mladec1).

**Fig 3:**
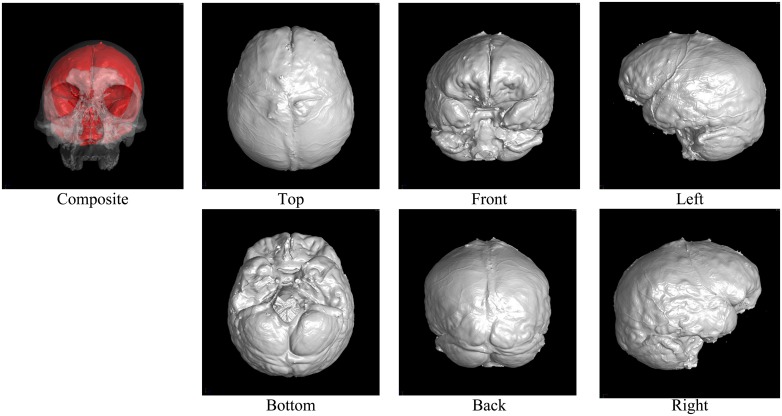
Results of endocast extraction (KUMA3008).

**Fig 4:**
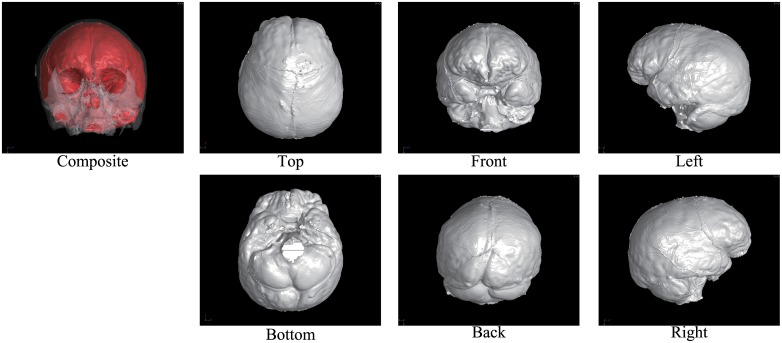
Results of endocast extraction (KUMA3147).

**Fig 5:**
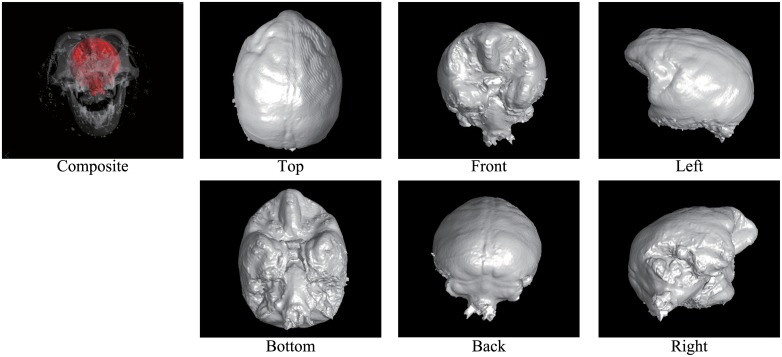
Results of endocast extraction (M15).

**Fig 6:**
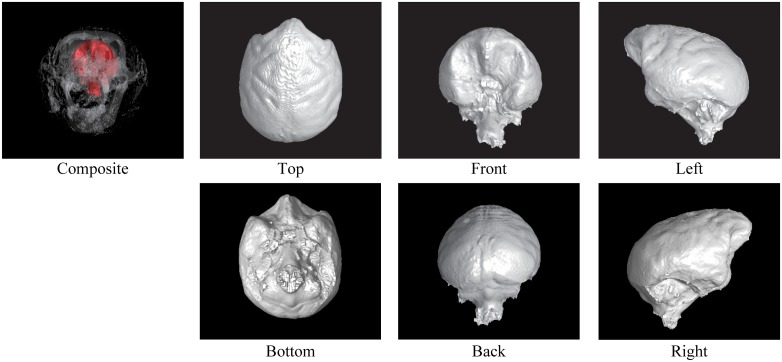
Results of endocast extraction (M16).

Figs 2 through 7 show the results of the endocast extraction. Although imprints of sulci and gyri are not usually identifiable on the endocasts extracted from adult human crania, they are known to be more pronounced in macaques [13]. Our results demonstrated that identification of cortical features from the endocast morphology may be possible for macaques (Figs 5 and 6) but not for adult humans (Figs 3 and 4).

**Fig 7:**
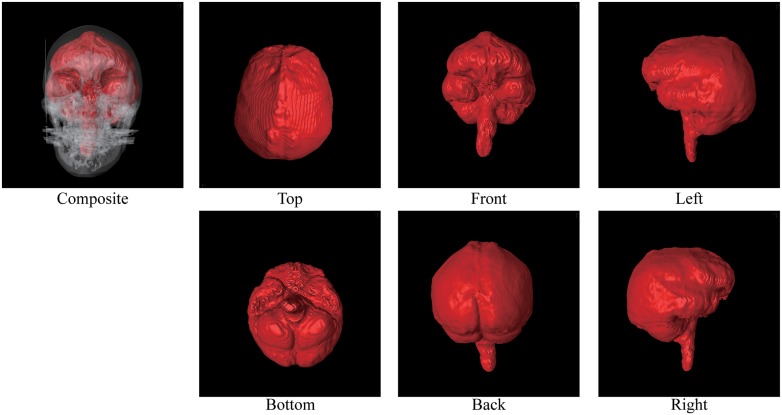
Results of endocast extraction (CT Head).

### Discussion

In the present study, we developed an automatic method for extracting the endocranial surfaces from CT images, in order to facilitate morphological analyses of fossil endocasts. As shown in Figs 2 through 7, the proposed method can extract only the endocranial surfaces from the CT images of human crania while other parts are not polygonized. In particular, surface bumps on the endocranial surfaces are well preserved for all examples. This is because the polygonization method used in the present study inherits sub-voxel accuracy from the marching cubes algorithm. Since other reconstruction methods also use this for polygonization, the result surfaces by other methods must be same, if the same threshold is given. In addition, the surface is guaranteed to be a closed two-manifold surface. These properties enable very efficient quantitative analysis and post-processing, because commonly used geometry processing tools assume that the input shape is manifold. Due to these two properties, the proposed method is easily combined with other geometry processing (e.g., mesh simplification [29]).

Note also that the proposed method can also handle tilted models. For example, the M15 model (Fig 5) is largely tilted. Although conventional slice-by-slice contouring is difficult, the proposed method can extract the endocast of such a tilted model because the computations are done in 3D.

**Fig 8:**
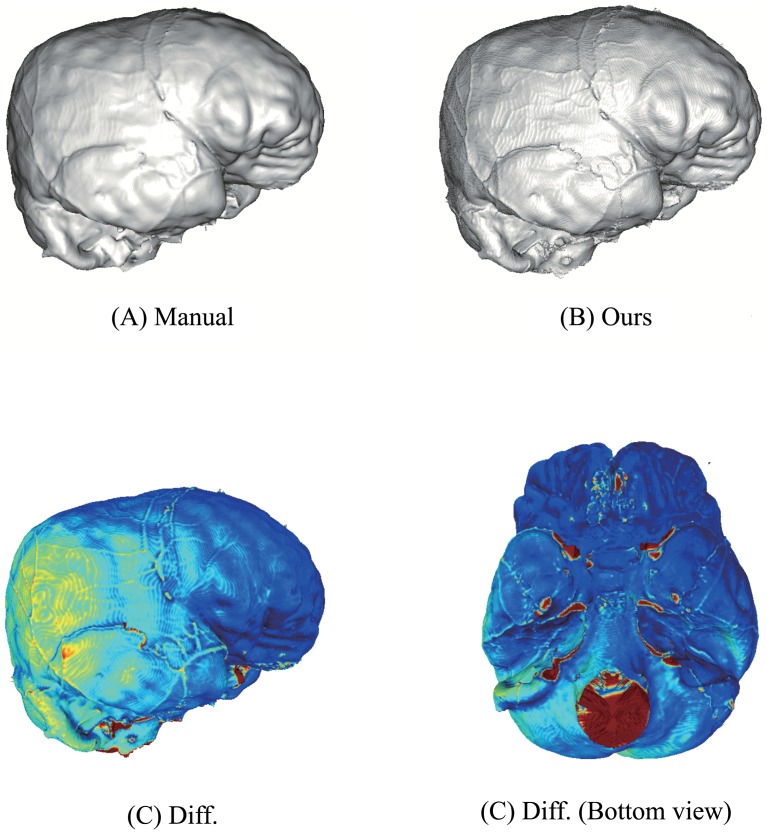
Manually obtained mesh and mesh obtained using the proposed method. The color maps in C and D show the differences between the manually obtained mesh A and the mesh obtained using the proposed method B (Blue: 0 [mm], Green: 0.5 [mm], Red: ≥ 1.0 [mm]).

**Fig 9:**
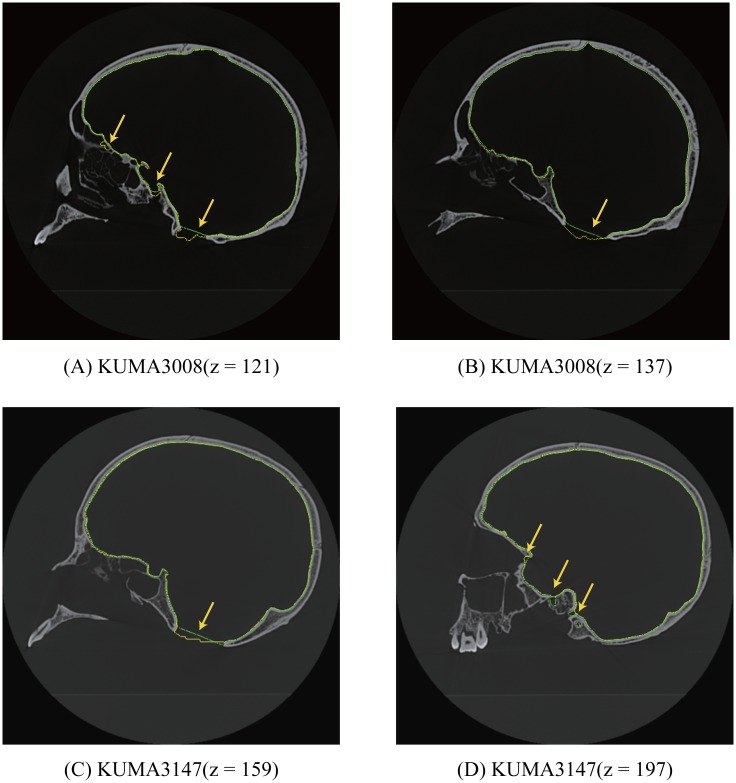
2D comparison of cross-section of CT images, polygonal models by manual operation (green) and our result (yellow). Yellow arrows show significant differences of polygons.

We compared the results obtained using the proposed method with those obtained by manual operations. Fig 8 shows the results for KUMA3147 obtained by manual operations [15] (Fig 8A) and by the proposed method (Fig 8B). According to [15], the results shown in Fig 8A was created using medical imaging software (Analyze 9.0; Mayo Clinic, Biomedical Imaging Resource, Rochester, MN, USA) and reverse engineering software (RapidForm 2006; INUS Technology, Seoul, Korea). The total working time for polygonization was two hours. Note that the polygonal models by [15] are pose-normalized, and we applied a shape registration method to them for evaluation of geometric difference. We confirmed that no significant difference could be found in either of the models. except for the filled regions such as foramen magnum (Fig 8C and 8D). The quantitative difference is very small (0.14 [mm] on average), and the maximum difference appears foramen magnum because these holes are filled in different criteria. Fig 9 shows cross-sections of the CT image and the polygon models. These images show that our segmentation method sometimes expands outside around larger holes. This depends on distance field used in watershed computation. On the other hand, expansion can also be observed in the polygonal models by manual operation as shown in Fig 9D. These differences show that clear criterion for filling these holes do not exist. However, our method provides consistent criterion for segmentation, hence any operators can automatically compute equivalent results from CT images.

**Fig 10:**
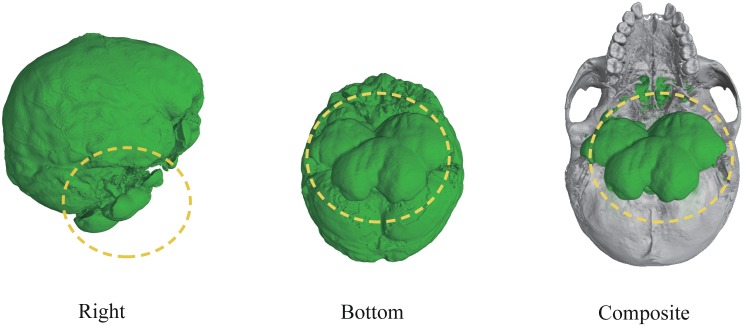
Result of endocast extraction using a level set method. The cranium and extracted endocasts are drawn in gray and green, respectively.

We also compared the present results with those obtained by a level set method using itk-SNAP [30], a popular segmentation tool in medical imaging. Fig 10 shows a result for KUMA 3008 model. As the figure shows, the extracted region protruded from the foramen magnum (yellow dotted circles) because the CT values of the background and the endocast region are similar. In order to overcome this problem, initial seed points must be carefully determined, but appropriate determination of the seeds is not easy and usually time-consuming.

The proposed method requires two major parameters in order to extract endocast models. The first parameter is the structural element size, or the radius of the sphere, used in the morphological closing in the binarization step. This is required for filling the small cavities in the cranium and the extent of the bottleneck will be a guide for parameter tuning. We used *r* = 6 [voxels] for all experiments. The other parameter is the isovalue for the endocranial surface. The isovalue is a common parameter for creating polygon data from CT images. The best threshold can be easily estimated by volume rendering software.

**Fig 11:**
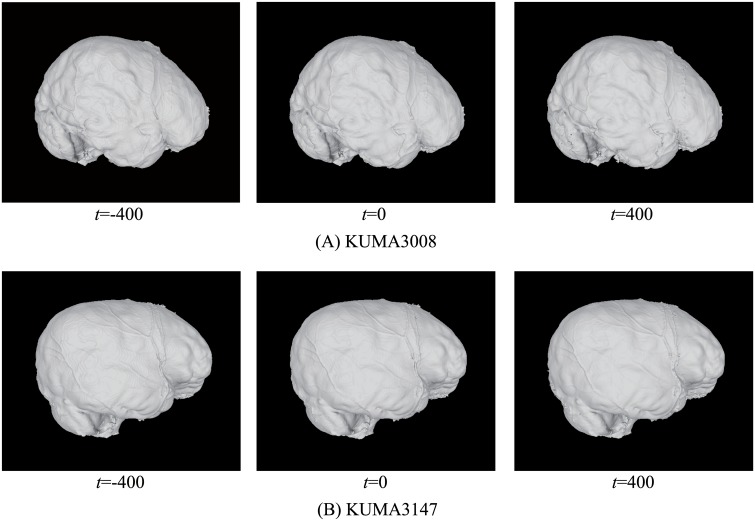
Results for KUMA3008 and KUMA3147 obtained using different thresholds.

In addition, the endocranial surface is robust to the variation of isovalues. Fig 11 shows the results for KUMA3008 and KUMA3147 obtained using different CT values, namely, -400, 0, and 400. No clear geometrical differences were observed between these results. The geometric differences between these models are approximately 1 voxel pitch of the CT images (0.28 ± 0.37 [mm] (KUMA3008) and 0.23 ± 0.43 [mm] (KUMA3147)).

The proposed method can also be applied to non-human primates. Figs 5 and 6 showed the results for the crab-eating monkey (*Macaca fascicularis*) models. These models have smaller endocasts, and automatic initial seed assignment failed in both experiments. Thus, the endocast is not always the largest empty space in the crania of non-human primates. For these examples, we provide an alternative approach to the initial seed assignment. Given the binary image and its distance field, we binarize the distance field by the threshold $\hat{t}$. The objective of binarization is to obtain two connected components used for “other” and “endocast” labels. The guideline for $\hat{t}$ is to fill all the bottlenecks connecting to the endocast space. We used $\hat{t}=10$ [mm] for the M15 data and $\hat{t}=15$ [mm] for the M16 data. Note that this is not necessary for the extraction of human crania because the endocranial region is the largest empty space in the cranium.

The computation times of the experiments are summarized in Table 2. The experiments were conducted using a Windows PC with an Intel Corei7-3930K (3.2 GHz) processor, 64 GB of RAM, and an NVIDIA Quadro 4000 graphics processing unit. Although our implementation has not been yet fully optimized, the computation time was less than ten minutes for all examples. We expect the computation time will be improved by, for example, optimizing the graphics processing unit. Although the computation time directly depends on the resolution of the CT images, we believe that the computation time for other samples will not exceed those in the experiments because the cranium models are usually scanned using medical CT scanners and the sizes of the CT images must be similar.

**Table 2:** Computation time of the experiments.

Specimen ID	Time[s]				Num. Polygon
	Bin.	Seg.	Pol.	Total	
Mladec1	182.8	146.2	41.2	370.3	1,137,492
KUMA3008	160.8	123.0	34.2	318.0	954,464
KUMA3147	150.5	99.0	34.9	284.4	985,560
M15	92.0	105.6	23.3	220.9	186,640
M16	95.7	106.2	24.1	225.9	193,428
CT Head	8.9	7.1	3.7	19.7	143,504

Bin.: Binarization, Seg.: Segmentation, Pol: Polygonization

The proposed method has three major limitations. First, the quality of the polygons largely depend on the results of binarization. Since the CT values of the thin part of the skull will be smaller than expected, it is hard to determine a good threshold and the binarization results may create unexpected voids. Although the proposed polygonization scheme may fill such defects as it is, the scheme should be improved in the future. The second limitation is how to define the boundary surfaces of canal structure. The last limitation is that the proposed method may fail when the assumption that the endocast is the largest empty space in the CT images does not hold. In such cases, other empty regions may be extracted.

## Conclusion

We have presented a method for computing endocranial surfaces from CT images of crania. One of the primary contributions of the present study is to automate endocast extraction using volumetric image analysis technology. The experimental results revealed that the proposed method could extract endocranial polygons from CT images of human crania within ten minutes using a common desktop PC. We expect that the proposed method will accelerate morphological analyses of fossil crania, such as the analysis of individual differences and inference of brain shapes.

The proposed method has the potential to extract other cavity structures in the human body (e.g., sinuses). In the future, we would like to extend the proposed method so that other anatomical features can be extracted. As such, we need to introduce other criteria in order to extract target features. In addition, we would like to address some limitations discussed in the previous section in order to allow accurate extraction of cranial surfaces.
